# Preoperative small pulmonary nodule localisation using hookwires or coils: strategy selection in adverse events

**DOI:** 10.1186/s13019-023-02301-6

**Published:** 2023-07-24

**Authors:** Tao Zuo, Zhaoming Gao, Tao Zhang, Bing Wen, Baojun Chen, Ping Jiang

**Affiliations:** 1 Department of Thoracic Surgery, Tongji Medical College, The Central Hospital of Wuhan, Huazhong University of Science and Technology, Wuhan City, Hubei Province China; 2 Department of Thoracic Surgery, Binzhou People’s Hospital, Binzhou, China; 3 Department of Neurosurgery, Tongji Medical College, The Central Hospital of Wuhan, Huazhong University of Science and Technology, Wuhan City, Hubei Province China; 4 Department of Lung Cancer Surgery, Tianjin Medical University Cancer Institute and Hospital, Huanhu West Rd, Tianjin, China; 5 Department of Ophthalmology, Zhongnan Hospital of Wuhan University, Wuhan, Hubei Province 430071 China

**Keywords:** CT-guided localisation, Ground-glass nodule, Lung cancer, Video-assisted thoracoscopic surgery

## Abstract

**Objective:**

This is a retrospective study of adverse events associated with preoperative computed tomography (CT)–guided hookwire or coil localisation. We analysed the experience and process flaws in resecting ground-glass nodules (GGNs) through video-assisted thoracoscopic surgery (VATS) and determined the remedial strategy.

**Methods:**

Adverse events were evaluated in 40 patients with 45 GGNs who underwent CT-guided hookwire or coil localisation before VATS. For lesions not successfully marked or detected, palpation, resection of the highly suspected area, segmentectomy or lobectomy was performed.

**Results:**

Among all adverse events, 15 were dislodgement of the marking materials, 5 were breakaway of the marking materials, 7 were > 2 cm distance between the lesions and the tips, 3 was marking material across the two adjacent lobes, 15 were pneumothorax and 2 were certain parts of marking materials stuck into the chest wall. All GGNs were resected successfully. 20 lesions were detected by palpation. 9 GGNs were discovered after the resection of highly suspected areas. Segmentectomies and lobectomies were performed directly on 7 and 9 GGNs, respectively.

**Conclusions:**

When adverse events occur, a second intraoperative localisation, by resecting the highly suspected area either through non-anatomical resection (wedge resection) or anatomical resection (segmentectomy or lobectomy) using the VATS should be considered the alternatives for GGNs localization.

## Introduction

In recent years, low-dose computed tomography (CT) has played an important role in the identification of pulmonary nodules in general [1]. Individuals with ground-glass nodules (GGNs) < 3 cm in diameter have better outcomes, with 5-year survival rates as high as 60–80%.^2^ To obtain a definite diagnosis of a GGN with high clinical suspicion of lung cancer on chest CT, confirmation of malignancy must be established by either needle biopsy or nodule resection. Video-assisted thoracoscopic surgery (VATS), a less-invasive type of thoracic surgery, can provide better evidence for the diagnosis of GGNs, while at the same time, active radical treatment should also be taken [2, 3]. However, if the GGN is ≤10 mm in diameter or is > 5 mm away from the pleural surface, it is incredibly challenging for surgeons to palpate these lesions [4].

A number of localisation techniques for subcentimeter-nodule have been devised to detect lesions and reduce the unnecessary loss of lung tissue during the surgical process. Metallic hookwire or microcoil localisation under CT guidance is the most widely used localisation technique worldwide [5]. It is also a safe and effective technique for preoperative localisation and increases the success rate of VATS [6–8]. However, adverse effects may occur, including puncture-related complications, complications of localisation and unsuccessful excision, do occur. We collected these adverse events with the aim of determining appropriate remedial measures.

## Patients and methods

The institutional review board of our hospital approved the present retrospective study. We obtained data by collecting electronic medical record systems and imaging systems.

### Definition of adverse events

Adverse events were defined as follows:

1. Puncture-related complications: pneumothorax, haemorrhage, air embolism, acute pain.
2. Complications of localisation: unhooking or dislodgement.
3. Unsuccessful excision: No lesion detected in the excised lung tissue due to unhooking or dislodgement.

### Patients

A total of 440 patients underwent the preoperative procedure of CT-guided hookwire and/or coil localisation were evaluated in this study between 1 and 2018 and 1 October 2020. Selection criteria were based on at least one of the following CT findings: lesion diameter ≤ 10 mm, no pleural indentation and pure GGN or a lesion mostly composed of GGNs. For patients with multiple GGNs, only those who underwent the adverse preoperative procedure of CT-guided hookwire or coil localisation were recorded in this study.

### Radiologic localisation procedure

All CT-guided localisations were performed on the day of VATS surgery. The hookwire set (275S090102; Pajunk GmbH Medizintechnologie, Geisingen, Germany) was composed of a calibrated cannula (21-gauge, 100-mm long) and a calibrated wire (20-cm long). The size, location, shape, number and surrounding tissue of the lesion were analysed before surgery based on the preprocedural CT images. A localized CT scan was performed in the area where the GGN was most likely located. A puncture site was forecasted, and an optimal trajectory was designed between the skin and the edge of the lesion. After the skin disinfection, administration of local anaesthesia and calibration of the cannula needle, the hookwire with the cannula needle was inserted through the skin and pulmonary parenchyma to reach the edge of the lesion. The thorn of the hookwire was then released, and the cannula needle was pulled out. A CT scan was repeated to ensure that the thorn was around the GGN and that the invasive manipulation did not lead to complications. The trailing end of the hookwire was covered by sterile gauze.

The coil localisation equipment included an embolisation coil (MWCE-35-5-4, Cook Inc., Bloomington, IN, USA), Chiba biopsy needle with 10-mm graduations on the needle shaft (DCHN-18-15.0, Cook) and Radifocus guide wire (RF*GA35153M, Terumo Corp, Shibuya, Tokyo, Japan). The procedure process was similar to that given above.

### Surgical procedures

Patients received general anaesthesia with double-lumen endotracheal intubation and were placed in the lateral position. Povidone–iodine was applied to the patient, who was wrapped in a sterile drape. The procedure of VATS resection required one (or two) 10-15 mm thoracic port(s) for the thoracoscope as well as the endoscopic medical apparatus and instruments. The trailing end of the hookwire was pulled into the chest and then folded, so as to avoid stabbing the organs. The wire or coil was followed by sponge forceps to detect the lesion during the procedure. The lung tissue was clamped, and the lesion was sequentially resected by staplers. The resected hookwire or coil and lung tissue were packed into sterile gloves to prevent metastatic implantation of malignant disease and were withdrawn from the chest via an intercostal incision. Examination of removed lung tissue to confirm marker and tumor integrity. When the lesion was not successfully localised, palpation with thoracoscopic instruments was first performed to localise it. When the palpation failed, resection was attempted on the highly suspected area or a segmentectomy or lobectomy was performed. All resected lung specimens were immediately sent for frozen section examination. If the pathological result was benign or indicated primary lung cancer, a chest tube was inserted after bleeding and air leak were excluded. If the diagnosis was infiltrating carcinoma, a segmentectomy or lobectomy and a lymphadenectomy or systematic lymph nodal sampling were carried out and if necessary, another thoracic incision was made to facilitate the subsequent thoracoscopic resection. Otherwise, if the result suggested a metastatic tumour following wedge resection, the procedure was terminated until a multidisciplinary treatment scheme was set up.

### Pathological diagnosis

Pathological diagnosis was classified according to the International Association for the Study of Lung Cancer/American Thoracic Society/European Respiratory Society International Multidisciplinary Classification of Lung Adenocarcinoma [9].

### Data analysis

We performed the statistical analysis using commercially available statistical software, IBM SPSS Statistics software version 20.0 (IBM Corp., Armonk, NY, USA).

## Results

Localizing adverse events occurred in 40 patients.The occurrence rate was 9.09%. 45 GGNs were detected on lung cancer screening CT in these patients; 17 patients were found due to symptoms of cough or sputum, 23 patients incidentally detected on CT. This study included 19 men and 21 women, with a mean age of 48 years (range, 28–72 years). The mean number of pack-years was 20.7 (range, 10–35). Of the 40 patients, two had a history of cancer, three had a history of hypertension, three had diabetes and two had tuberculosis (Table 1).

**Table 1:** Characteristics of 45 patients who underwent an unsuccessful preoperative procedure of CT-guided hookwire or coil localization and VATS.

Characteristic	No. of patients
Age(years)	48(range,28–72)
Gender	
Man	19
Female	21
First symptoms	
Founding by physical examinations	23
Cough or sputum	17
Cancer history	
Yes	2
No	38

Among the 40 patients, one had two lesions to localise and one had four lesions to localise, which were situated in various segments of the left lung. The GGNs were located in the right upper lobe (n = 10), right middle lobe (n = 6), right lower lobe (n = 9), left upper lobe (n = 11) and left lower lobe (n = 9). The diameter of the lesions ranged from 4.0 to 12 mm (mean, 8.5 mm). The distance of the lesion from the pleural surface (including the interlobular pleura and mediastinal pleura) ranged from 1.5 to 55 mm (mean, 15.6 mm).

**Fig. 1:**
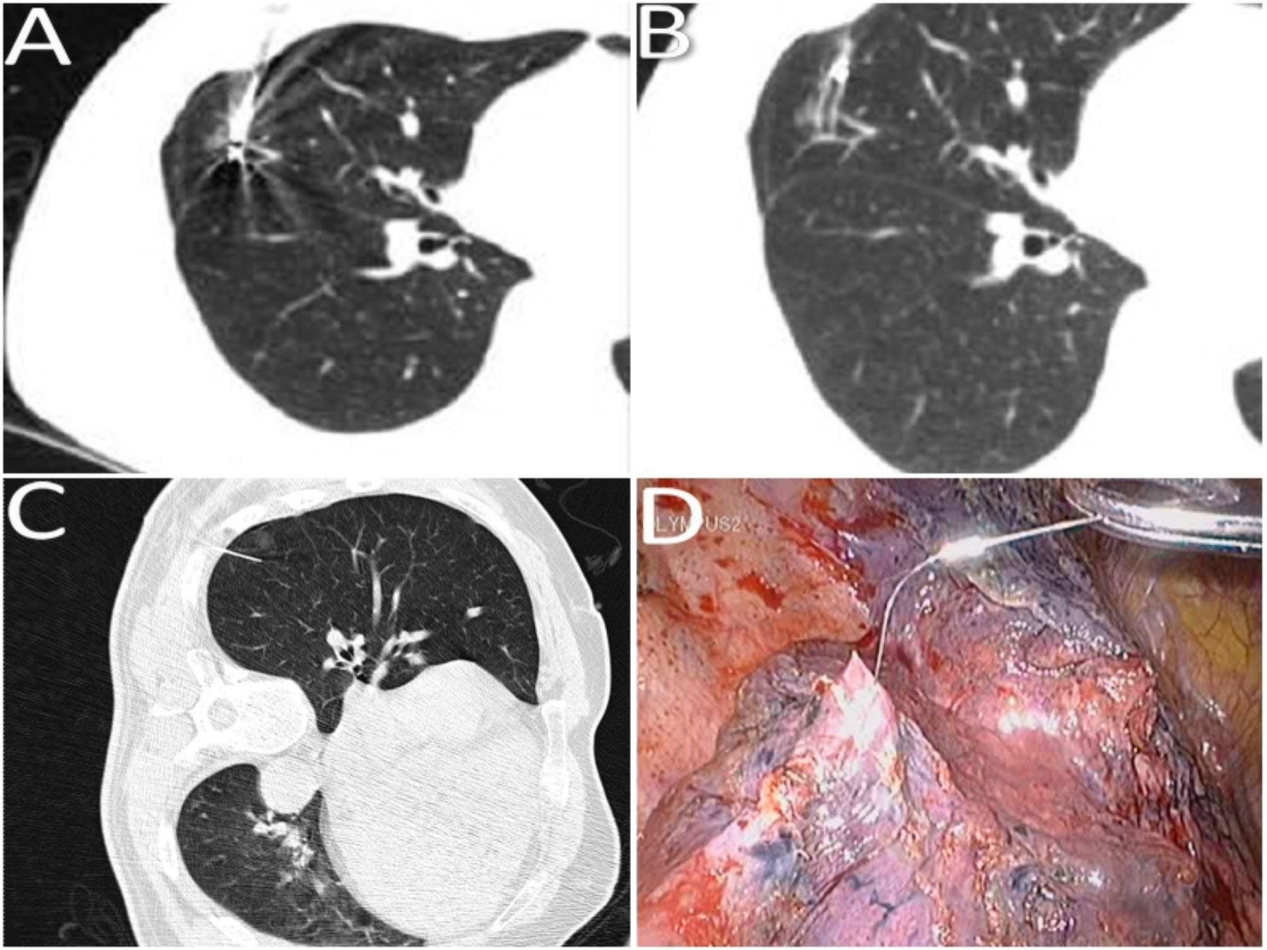
Images of lung computed tomography (CT) scan and surgical field for dislodgement of marking materials. **(A)** The coil was adjacent to a GGN after it was released. **(B)** Coil dislodgement occurred through the needle passage. **(C)** The tip of the hookwire was extended more than 3 cm into the lung tissue before the operation. **(D)** The tip of the hookwire was extended less than 1 cm into the lung tissue during an operation

**Fig. 2:**
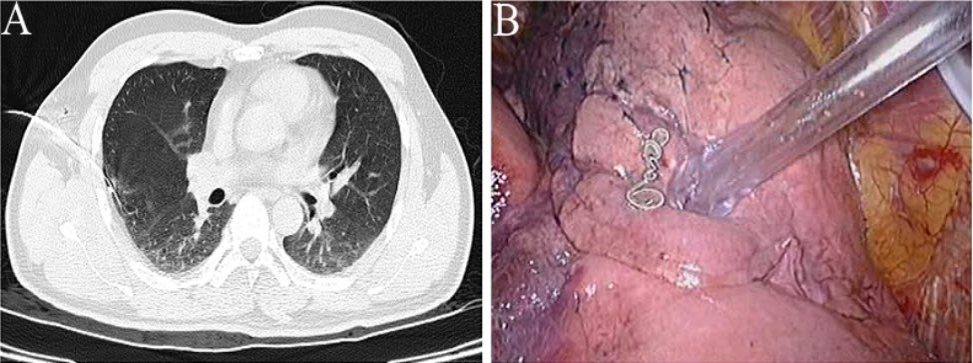
Images of lung CT scan and surgical field for the breakaway of marking materials. **(A)** A breakaway of the coil was observed after the trocar needle was withdrawn. **(B)** A breakaway coil was found in the pleural cavity during VATS

**Fig. 3:**
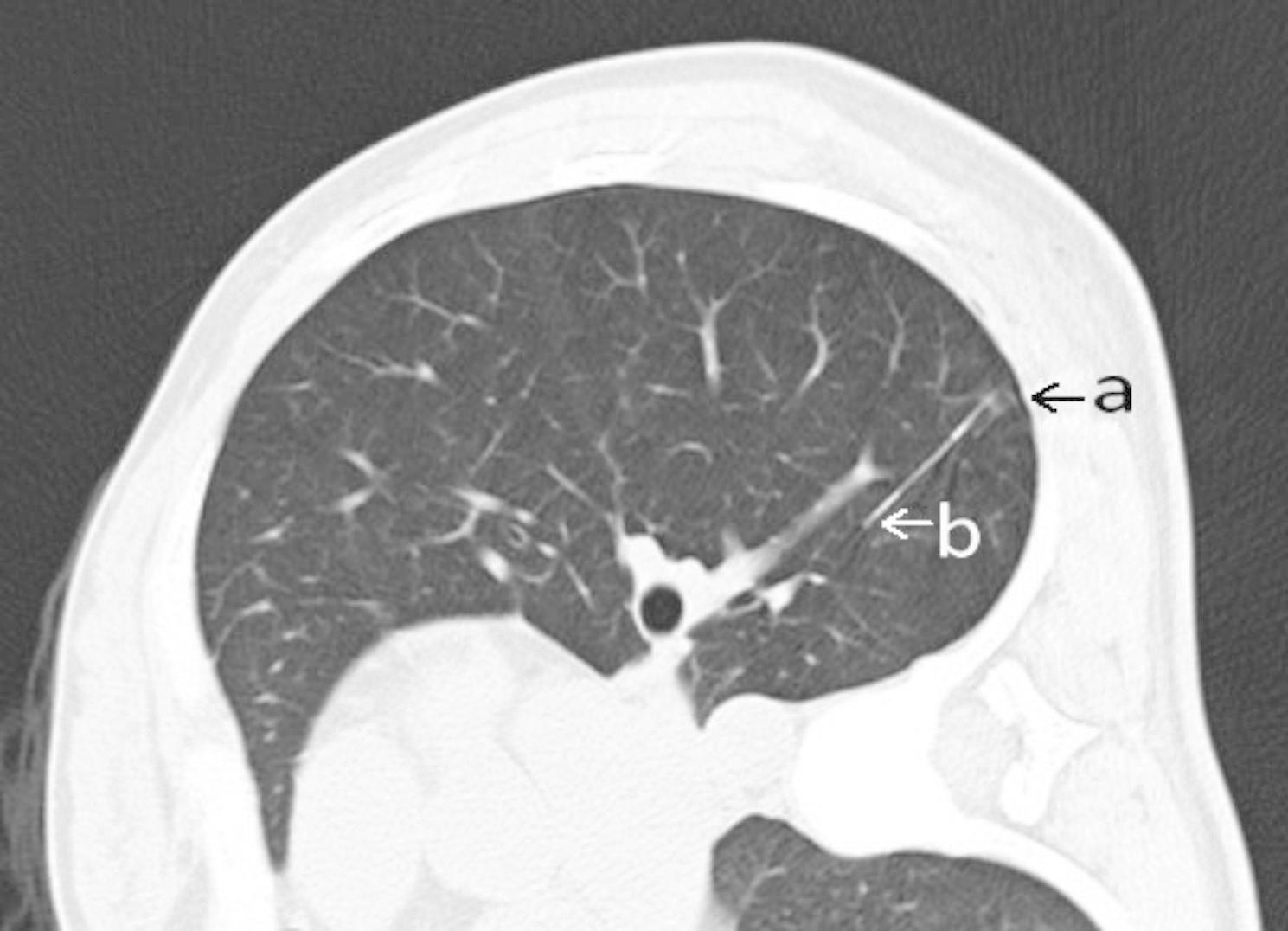
Images of lung CT scan for the distance between the lesion and the wire tip. As shown in the image, there was a distance of more than 2 cm between the lesion and the tip of the wire. (**a:** lesion, **b:** tip of the wire)

**Fig. 4:**
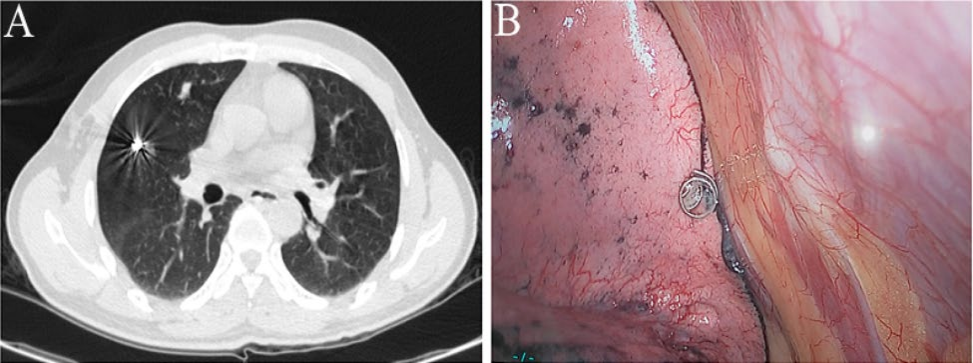
Images of lung CT scan and surgical field for coil localisation. **(A)** A GGN in the right middle lobe was localised with the coil. **(B)** The coil for lesion localisation was found in the upper lobe

**Fig. 5:**
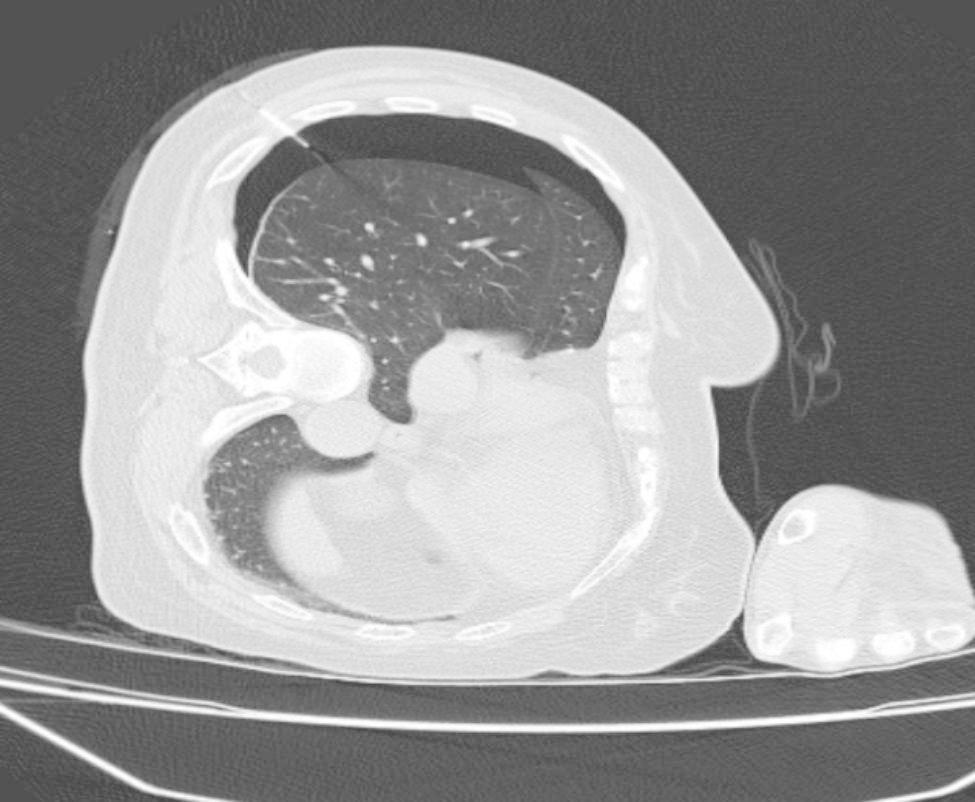
Images of lung CT scan for the development of pneumothorax. A mild-sized right pneumothorax, characterised by a banded area of very low density without lung texture, compression of the lung parenchyma and deviation of the mediastinum toward the contralateral hemithorax

**Fig. 6:**
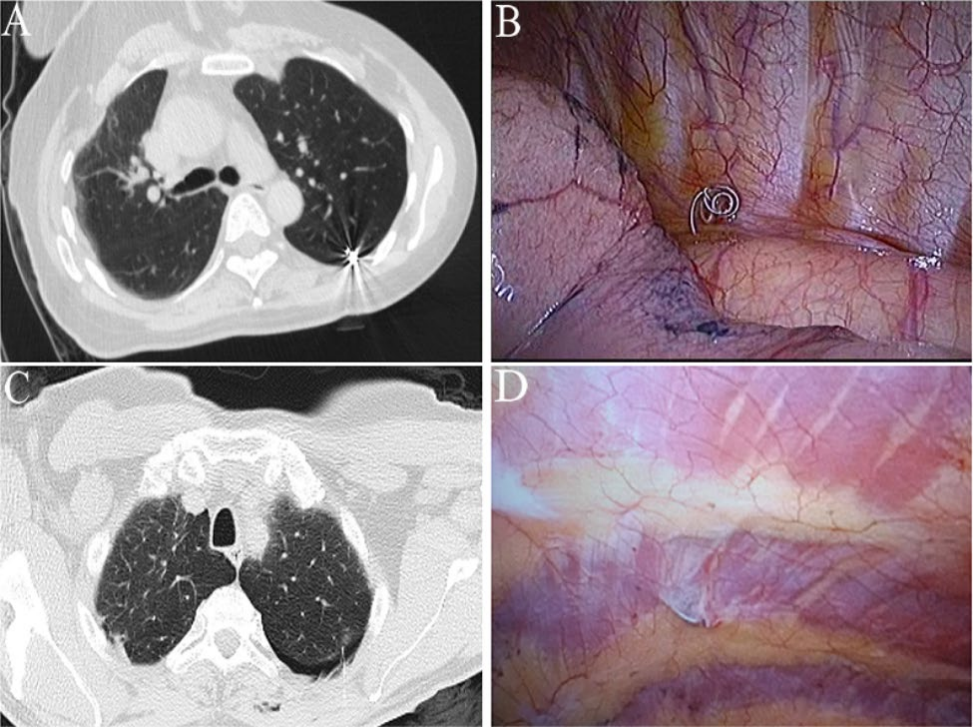
Images of lung CT scan and surgical field for placement and fixation of marking materials. **(A)** The coil was placed into a GGN adjacent to the pleura. **(B)** The coil was fixed to the chest wall. **(C)** The tip of the hookwire was placed into a GGN at the left lung. **(D)** The hookwire was fixed to the chest wall

**Fig. 7:**
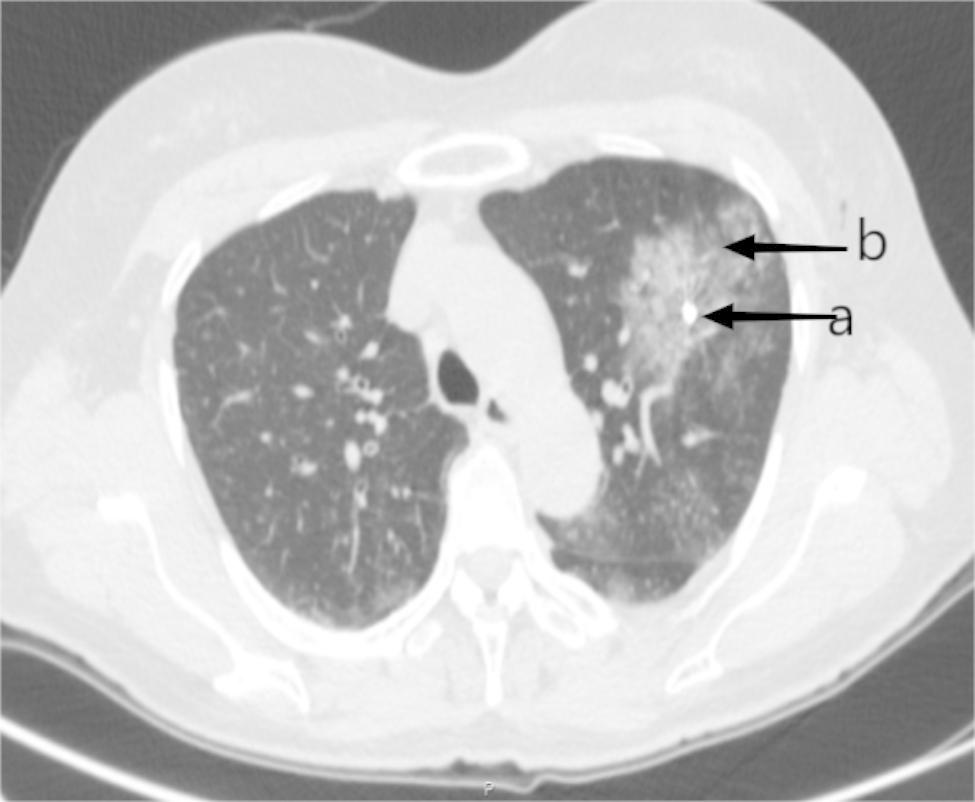
Images of lung CT scan for the development of pulmonary haemorrhage.(**a:** coil, **b:** pulmonary hemorrhage)

A total of 24 and 21 lesions were marked by hookwires and coils, respectively. As for the types of all unsuccessful localisation, 15 had dislodgements of marking materials (Figs. 1), 5 had breakaway of marking materials (Figs. 2), 7 had a distance of > 2 cm between the lesion and the tip of wires or coils (Figs. 3), 3 had the marking material in different lung lobes adjacent to the GGN (Figs. 4), 15 developed pneumothorax(Figs. 5) and 2 patients had certain parts of marking materials stuck into the chest wall (Fig. 6)(Table 2). No severe complications occurred after this procedure, but several grade 1 adverse events were observed(Common Terminology Criteria for Adverse Events Version 5.0). None of the cases with pneumothorax required a particular intervention, such as manual aspiration of air or chest tube placement.

**Table 2:** Characteristics of 45 GGNs from 40 patients who underwent an unsuccessful preoperative procedure of CT-guided hookwire or coil localization and VATS

Characteristic	No. of lesions
Marking material	
Hookwire	24
Coil	21
The types of unsuccessful localization	
Dislodgement	15
Breakaway	5
The distancemore more than 2 cm between the lesion and the tip of wires or coils	7
Marking material in different lung lobes adjacent to GGN	3
Pneumothorax	15
Marking materials stuck into walls	2
Remedies	
palpation	20
Resecting the highly suspected areas	9
Segmentectomy	7
Lobectomy	9
Histological diagnosis	
Interstitial fibrous tissue proliferation	4
Atypical adenomatoid hyperplasia	7
Adenocarcinoma in situ	8
Minimally invasive adenocarcinoma	10
Invasive adenocarcinoma.	16

All GGNs were successfully resected. 20 lesions were detected by palpation. 9 GGNs were discovered after the highly suspected areas of lung were resected. When the GGN could not be detected after a highly suspected area of lung, a lobectomy or segmentectomy was carried out.Segmentectomies and lobectomies were performed directly on 7 and 9 GGNs, respectively.

Frozen section histopathology provided adequate information for appropriate intraoperative management, as confirmed by subsequent permanent section analysis in all cases. Of 45 lesions, 4 were interstitial fibrous tissue proliferation, 7 were atypical adenomatous hyperplasia, 8 were adenocarcinoma in situ, 10 were minimally invasive adenocarcinoma and 16 were invasive adenocarcinoma.

## Discussion

In our study, we found a second localisation, intraoperative localisation, resection of the highly suspected area, or a segmentectomy or lobectomy can be successfully attempted using VATS for resection of GGNs after failure in localising lesions.

Pulmonary wedge resection using VATS is the most commonly used method available for the surgical treatment of GGNs < 1 cm in diameter. However, the probability of palpation failure in localising lesions can be as high as 63%.^10^ Thus, accurately localising the lesions is crucial.Traditionally, we can classify the localisation of lesions into three types [11, 12]. The first type is localisation with imaging modalities during thoracoscopy. This includes intraoperative ultrasonography [13] and CT fluoroscopy [14]. The second type is preoperative localisation with an injection of dyes [15], contrast media [16], radionuclides [17] or coloured adhesive agents [18]. The third type is preoperative localisation with hook wire or coil placement [19, 20]. The most popular localisation technique is CT-guided hookwire or microcoil localisation. Most studies published in recent years have reported high success rates of localisation with coils or hookwires, with rates ranging from 0.4–42%.^8,10,20,21^.New methods, sunch as injecting indocyanine green (ICG) under the guidance of electromagnetic navigation bronchoscope,may cost more money and requires more equipment [22].

### Common adverse events of localisation and their cause

Complications of coil and hookwire are similar [23, 24]. Dislodgement is the most common cause for operation failure. Iwasaki reported that wire dislodgement occurred in up to 20% of cases [25]. Among our occurrences of placement failure, 15 patients had dislodgement of marking materials, of which 10 and 5 were marked by hookwires and coils, respectively. According to Mullan et al [26]., a wire is generally dislodged at one of three times: during transportation of the patient to the surgical suite, during surgical deflation of the lung, or during resection, when the surgeon often applies gentle retraction to the wire. Coil dislodgement occurs less than wire dislodgement does, because its rough fibre coating induces coagulation and increases adhesion to the lung tissue, and its tension fixes it on lung tissue cracks. Nonetheless, a wide needle passage or tiny pulmonary elastic resistance still causes dislodgement. The coil is soft and pliable and causes less damage to lung tissue than the wire does when dislodged. Gagliano et al [27]. reported a case in which a displaced coil was uncoiled, causing less tissue damage when compared with hookwire for localisation in ex vivo goat lungs. Breakaway is a special type of dislodgement. The breakaway of marking materials was observed in 5 patients in our study. The most serious breakaway we encountered occurred when the wire tip was embedded in the chest wall. According to Seo et al [21]., the distance between the wire tip and pleural surface can be regarded as the only independent factor for successful localisation. When the distance is < 1 cm between the marking materials and pleural surface, the marking materials can break away from the lung due to the cutting force of the wire or the tension of the coil.

A distance of > 2 cm from the GGN to the tip of the wire or coil might have risk of failure for localization which occurred in 7 cases in this study. During the operation, the location of the GGN and safe resection margin cannot always be predicted. An unskilled operator or uncooperative patient urges to this kind of failure. In our case, it is generally due to pulsations in the cardiovascular system and abnormal respiratory rate.In addition, deeper lesions are more likely to have such adverse event.

If the tip of the wire or coil is located in different lobes of the lung adjacent to the GGN or across two adjacent lobes, difficulties will be encountered during the operation, and even normal lung tissue will be removed. A distance of < 1 cm between the marking materials and adjacent pulmonary fissure is an important risk factor for this kind of failure.

In contrast to dislodgement or breakaway, the entire or a portion of a hookwire or coil sometimes gets stuck in the chest wall when the GGN is in the vicinity of the pleural surface, and meanwhile, marking materials are released into the pleural cavity.

Another important reason for unsuccessful localisation is the occurrence of complications. Common complications include pneumothorax, haemorrhage, air embolism, acute pain The incidence rate of pneumothorax may account for nearly half of all complications of this procedure [8, 10]. Repeated puncture, thick puncture needle and large-diameter coil are the main reasons for pneumothorax. Generally, haemorrhage can be divided into two categories: pulmonary haemorrhage (Fig. 7) and haemothorax. Minor pneumothorax and asymptomatic bleeding are frequent during localization and usually do not require treatment. Air embolism usually occur in wires. And the fiber coating of coils reduce the risk of embolization [27].

### How to avoid failure of localisation

First, we need to determine the indications for CT-guided hookwire or coil localisation. Ciriaco et al [4]. concluded that preoperative CT-guided hookwire localization for pulmonary nodules is an effective technique which allows VATS resection of PN < 10 mm located > 15 mm from the pleural surface. Saito et al [28]. indicated that a linear function (i.e. depth = 0.836 × size − 2.811) could be used to differentiate between undetectable and detectable small peripheral GGNs and that preoperative hookwire marking for small peripheral GGNs should be considered for nodules in regions above those.Our previous research found that CT-guided microcoil placement is an effective method of marking GGO lesions that makes thoracoscopic wedge resection easier for the “blind areas” of the hook-wire technique, including the mediastinum-vicinity region, interlobar fissure-neighbouring areas and scapulae-shadowed areas [5].

For lesions with a distance of < 10 mm from the pleural surface, the tip of the wire or coil should be extended about 10 mm beyond the edge of the lesion. It is widely recommended that the pleural end of the coil or wire be removed from the chest wall via thoracoscopy. The tail end of the hookwire should not to be fixed when CT-guided hookwire localisation is completed, to avoid pulling to deflation of the lung. The coil should not have a thick diameter to avoid a wide needle insertion route. These measures can contribute to reducing the incidence of dislodgement or breakaway.

When a GGN hides in the inner side of the shoulder blades and blocks the route through which the needle goes, it is advisable to place a hookwire after the upper body posture is adjusted. However, shoulder blades change their positions in the lateral position, and pulling the wire during coil localisation can avoid this failure.

The patient must be induced to remain static and relaxed. Because lesions in the lower lung are easily influenced by respiration, respiratory coordination in patients with such lesions is thus of great significance. Avoiding repeated punctures and paying more attention to pulmonary vessels and airways will assist in preventing complications.

### What to do after an unsuccessful localisation

Under certain circumstances, despite an unsuccessful preoperative CT-guided hookwire localisation, relocalisation will be attempted. Two types of localisations can be tried simultaneously [29]. Though multiple percutaneous puncture produced a significantly higher incidence of pneumothorax and hemorrhage, the localizations were clinically feasible and safe [30, 31].

Lung nodules can be localised during surgery after every effort is exerted before operation. Palpation is the easiest way to detect GGNs during VATS. Suzuki et al [11]. demonstrated that in cases of lesions of ≤ 10 mm in size, if the distance to the pleural surface is > 5 mm, the probability of failure to detect the lesions is > 50%; when the distance is > 10 mm, the failure probability is 100%. However, we detected 20 lesions by palpation. Finger palpation is the simplest method. Radiographic findings on preoperative CT images puncture site and the position of the unsuccessful localisation materials in the lung all help to detect small pulmonary lesions during thoracoscopic exploration. The success rate of palpation by which lesions were detected can be verified. Based on the radiographic findings on preoperative CT images, puncture site and the position of unsuccessful marking materials in the lung, detecting the highly suspected area of lesion in lung tissue is feasible. A point we raise with regard to the detection of lesions by palpation is to clamp this area with sponge forceps and run a finger over this area along a straight line. In this way, even a slight difference in the sense of touch can be felt. However, this technique does not usually work if the lesion is located deep in the lung parenchyma.

In cases in which palpation failed to localise the nodule, Suzuki et al [18]. chose to convert to thoracotomy. Resection of the highly suspected area of lesion in the lung tissue is also commonly used. Some newly designed methods or tools for the detection of pulmonary lesions have been considered effective during thoracoscopy. Ohtaka et al [32]. described that O-arm is an intraoperative imaging device that can provide CT images and that the positional relationship between the lesion and needle marking will be determined based on these O-arm CT images. Barmin et al [33]. designed a new tactile mechanoreceptor, with the help of which the surgeon can see the border between normal and high-density tissue in the inspected area. Okusanya et al [34]. intravenously injected indocyanine green 24 h before surgery and claimed that, during lung resections, intraoperative near-infrared imaging can be used to detect GGNs that are poorly visualised on CT and difficult to discriminate on finger palpation. All of these new methods are considered to be additional tools for facilitating intraoperative localisation and surgical resection of nonpalpable lung lesions. Segmentectomy or lobectomy should also be considered during surgery after every traditional effort has been exerted preoperatively.

Radiotracer-guided localization utilizes radioisotopes [35], such as technetium 99, have a history of highly successful resection rates. Electromagnetic Navigation Bronchoscopy (ENB) utilizes CT imaging to create a 3-dimensional (3D) virtual image [36],which can identify the airways proximal to the lesion through simultaneous virtual and bronchoscopic imaging, allowing the flexible bronchoscopy catheter steering ability to localize the target. Other novel systems such as robotic bronchoscopy [37], Ion Endoluminal System [37], and the SCOUT system [38] show great potential to reduce the limitations of current localization methods, and may become viable alternatives for lung resection, and can be applied to both minimally invasive VATS and robotic surgery. But they requires special equipment, advanced training and expertise, and is not viable for low-income or rural hospitals.

Wu et al [39]. reported that 3D navigation combined with anatomic segmental pulmonary resection avoids the adverse factors of puncture, and can replace puncture localization for GGN. Based on digital reconstruction and 3D printing technology, Li et al [40]. used preoperative HRCT image data of patients to prepare personalized simulation localization model of pulmonary nodules, which can be used to guide non-invasive real-time localization of pulmonary nodules during surgery.These methods do not require additional examinations, surgical equipment, or additional positioning time prior to surgery. And there are no complications such as puncture, trauma, pain, pneumothorax, hemothorax, etc. 3D assisted surgery may be a new direction for the localization of pulmonary nodules, but the accuracy and efficiency need to be further verified in multicenter applications.

While paying attention to these innovations, we should clearly realize that puncture localization of pulmonary nodules will remain a mainstream method for a long time in the future, especially in underdeveloped countries and regions and rural hospitals. This is mainly due to its simplicity, practicality, effectiveness and low cost.Therefore, it is of certain significance to summarize the experience of adverse events in this study.

We acknowledge some limitations of this retrospective study. First, most of the lesions in the cases we included were subcentimeter nodules. The smaller the lesion to locate, the more difficult it will be to do so. In addition, our study was not designed to compare the failure rate, cause of localisation failure and the method of amending after unsuccessful localisation. Moreover, we have become aware of some potential technical biases in our study. Despite these limitations, the results of this study contribute to additional significant experience on localisation failure, and we conclude that, after an unsuccessfully preoperative procedure of CT-guided hookwire or coil localisation, a second localisation, intraoperative localisation, resection of the highly suspected area, or a segmentectomy or lobectomy should be an option step by step VATS for resection of GGNs.

## Data Availability

The datasets used and/or analyzed during the current study are available from the corresponding author on reasonable request.

## Abbreviations

3D: 3-dimensional
CT: Computed tomography
ENB: Electromagnetic Navigation Bronchoscopy
ICG: Indocyanine green
GGN: Ground-glass nodule
VATS: Video-assisted thoracoscopic surgery
